# Supplementary material for: Capturing and analyzing pattern diversity: an example using the melanistic spotted patterns of leopard geckos
Source: PeerJ. 2021 Sep 10;9:e11829. doi: 10.7717/peerj.11829 (PMC8436963; doi:10.7717/peerj.11829)
Supplement: Supplemental Information 1 [file peerj-09-11829-s001.docx]

# Appendix

## Computation of p-values for Figures 7 and 8 and Table A1

We used nonparametric test procedures to compute $p$-values for Figure 7 and 8 and Table A1. For instance, for the comparison of within-individual and between-individual distances of the front legs,, let $\mu_{within}$ denote the mean within-individual square distance between the two front legs, i.e. the mean square distance in pattern space of the two front legs of the *same* animal. Let $\mu_{between}$ denote the mean between-individual square distance, i.e. the square distance between front leg patterns of two *different* animals. Fourteen geckos had patterns on both front legs. To compute $p$-values for the test of the null hypothesis $\mu_{within}\geq\mu_{between}$, we generated samples by pairing each of the 14 geckos with another, different gecko in such a way that each of the 14 were chosen exactly once as a ‘partner’. Each such sample corresponds to a permutation of the geckos without fixed point, also called a derangement. For each sample, we chose one of the two front legs of the first partner gecko and one front leg of the second partner gecko. We computed the sample means of the square distances of those two front legs. We repeated this procedure 10,000 times, choosing a randomly selected derangement each time. The reported $p$-values are the proportions of samples in which the mean sample square between-individual distance was equal to or exceeded the mean square within-individual distance..

Similar permutation approaches were used for the other tests in Figures 7 and 8 and Table A1.

## P-values of correlation coefficients for Tables 4 and 5

The p-values for the correlation coefficients for Tables 4 and 5 were computed with matlab’s *corrcoeff* function (MATLAB R2016b).

## Quantification of measurement error

We took four independent photos of each body part, where the animal was picked up and rearranged for each repetition so that the four measurements would be independent. Although efforts were made to minimize the measurement error by standardizing the overhead lighting and arranging the geckos on a template, measurement error was introduced by slight differences in the rotation and placement, especially for the limbs and tail. While the geckos were sedated, the limbs and tail could be arranged carefully; however, even the muscle tone throughout the limb and tail was variable, affecting the overall contour of the body in subtle ways. Small differences in angles along the contour of the body result in subtle lighting differences that would affect the exact contour of a spot or even whether a spot met the threshold of a spot in borderline cases (see Figure S1, Panels B, D and E for examples of patterns with spots that vary in a continuous way in intensity). Larger differences occur between measurements depending on whether connecting “threads”, which were often of borderline intensity, would meet the requirement for the threshold (several of these connecting threads are shown with red arrows in Figure S1).

In the first approach to characterize the measurement error, we consider body part patterns as points in 14-dimensional phenotype space and use distances between them. We determined the ratio of the mean distance of the four measurements of the same body part of the same animal from their centroid relative to the mean between-individual distances for that body part, or (for front or back legs) the corresponding within-individual differences. This is shown in Table A1 by means of the Mahalanobis distance and the Developmental Noise distance. In all cases, the mean within- or between-individual distances were significantly greater than the mean distance due to the measurement error, with factors varying from 2.2 (back leg, within-individual distance relative to measurement error, Mahalanobis distance) to 86.1 (tail, between-individual distance relative to measurement error, Developmental Noise distance). Table A1 also makes it possible to compare the absolute measurement errors of the different body parts (top row). The back legs had the largest average measurement error, 2-3 times that of the front legs. The front legs had similar measurement errors as the trunk, with much smaller errors for the head and tails.

A two-way ANOVA test where the two factors are “sides” (S; fixed) and “individuals” (I; random) (Palmer and Strobeck, 1986; Merila and Biorklund, 1995; Breuker et al., 2006) was performed separately for both pairs of front legs and pairs of back legs for each of the 14 indices. The data sets were all individuals with patterns on both front legs, or on both back legs, respectively, regardless of morph. The ratio $F=MS(S\times I)/MS(E)$, where $MS(E)$ is the mean sum of the squares for the error, can then be used as a measure of the relative size of the measurement error. Results are summarized in Table A2. For each index, an F-test yielded that nondirectional asymmetry is making a significant contribution to the variation observed relative to measurement error. The F-values ranged between 2.3 and 96, with a median value of 6.8, meaning that the measurement error made up between 43% and 1% of the observed variation with the median value corresponding to about 15%. We also tested whether directional asymmetry was present via a two-sided t-test. Except for the indices PL and EED for the back legs, this was not the case (note that the two p-values below 0.05 were 0.03 and 0.01, so given that we performed 28 tests of significance, this is fairly weak evidence for the existence of directional asymmetry even in these two incidences).
