# Supplementary material for: Capturing and analyzing pattern diversity: an example using the melanistic spotted patterns of leopard geckos
Source: PeerJ. 2021 Sep 10;9:e11829. doi: 10.7717/peerj.11829 (PMC8436963; doi:10.7717/peerj.11829)
Supplement: Supplemental Information 2 [file peerj-09-11829-s002.pdf]

Supplementary Material for Glimm. Kiskowski, Moreno and Chiari, "Capturing and analyzing pattern diversity: an example using the melanistic spotted patterns of leopard geckos"

Origin of geckos

Different morphotypes in the leopard gecko are obtained through controlled captive breeding aimed at selecting for certain characters related to body and/or eye color, spots vs. stripes and loss of pattern; an introduction to the different well established morphotypes and a general description of the "normal" morphotype vs. other more or less "pure" morphotypes can be found on several web sites of commercial gecko dealers, see e.g. Sykes (2004) and GeckoBoa Reptiles (2008).

All the geckos used in this study are of captive-bred origin and were obtained from different sources (see Table 1 for details) to ensure variation among individuals in the amount of melanistic patterns that exist for captive bred animals. No animal used in this study was purchased or obtained for their pattern, as we only requested geckos with a "normal" melanistic morphotype, which includes all geckos with a melanistic pattern on a yellowish or brownish background. While geckos with "normal" morph are more similar to the "wild type" pattern of this species - and some of the captive bred individuals may have some wild type in their blood line - this morph is generally very variable in terms of melanistic pattern (e.g., Figure A1 in the Appendix) and individuals are bred without specifically selecting for one or more characters in contrast to what happens with the "pure" and different morphs that can be created in this species (albino, tremper albino, melanistic, lemon frost, etc.). The advantage of using this approach - randomly selecting geckos with a "normal" morph obtained through captive-breeding or colleagues - is that by doing so we ensure having a very variable sampling of geckos for this general "normal" morph, as different people obtain them through breeding distinct parental individuals as long as they have the general "normal" pattern apparence.

Procedure for image acquisition

Geckos were anaesthetized using an open drop technique with cotton balls embedded with isoflurane. Anaesthetization of the geckos ensured that the animals were asleep while we took pictures for all the four datasets for each animal. Pictures were taken for the entire body of the animal - including the head and the tail if the entire animal fit into the frame - and for the two sets of legs (front and back) separately. Legs were held stretched out by two of us (NM and YC) and placed at the same angle from the body across picture sets. Legs were always positioned on top of the horizontal lines printed on the colored paper, while the body of the gecko was placed on the vertical line. Depending on the entire length of the animal, pictures of the tail were obtained together or separately from the picture of the main body. ID cards with information about the picture set were placed in the frame of all photos for use in identification during processing of images. A ruler was also placed in the frame of all the photos as a size reference. Photos were taken using a photography stand with the camera positioned directly at 12 cm above the gecko. Lighting was provided by the overhead lights in the room and by two additional lamps

that were attached to the stand and directed towards the geckos. We used a Canon EOS Rebel T6i camera with focal length of 18mm exposure time of 1/80, aperture of f/8, ISO-400, and auto-focus on. Settings were consistent across all photos and picture sets. To obtain pictures for different body parts (e.g., body vs legs), the animal was moved and then replaced every time for each picture set. We first obtained all the four sets for an animal before obtaining pictures for a new individual.

Description of patterns on different body part

Each of the four body parts (limb, trunk, head, and tail) presented different challenges so that the criteria for identifying melanistic spots were different for each type of body part. For example, the legs of the gecko were rounded and topographically complex with sharp angles due to the bones of the leg (see Figure S1, Panel G), whereas the head, trunk and tail were relatively flat. Thus the legs were unevenly illuminated and the algorithm for identifying the pattern of the legs included steps to brighten shadowed regions and darken bright regions. Shadows and glare were identified as contiguous areas of pixels that were darker or brighter than the mean pixel intensity at a spatial scale larger than the spatial scale of the spots. This method was effective since the melanistic spots on the legs were consistently smaller than the spatial scale of the leg contouring for all the gecko morphs. Spots identified by the algorithm almost always coincided with melanistic spots identified via eye inspection; only in a very few cases did the algorithm identify what looked like shadows as spots or vice versa (a comparison of photos and algorithm-identified spots for all patterns is added in the Supplementary Material). For the purposes of objectivity, consistency and reproducibility, the results of the algorithm were always used, even when there was a discrepancy between the algorithm and the pattern identified by eye. The trunk presented the challenge that dark crescent-shaped shadows of tubercules were difficult to distinguish from melanistic spots (see Figure S1, panel D). We found that these shadows were darker than melanistic spots in the blue channel and a satisfactory fraction of these shadows could be distinguished from the melanistic pattern by imposing additional blue channel criteria. Melanistic patterns varied considerably in their relative darkness, from gecko to gecko, body part to body part and even within a regional pattern (see Figure S1, Panels B, D, E and H). A qualifying melanistic pattern was identified by selecting pixels for each image that were sufficiently dark relative to the average pixel brightness of that image. The relative amount of darkness that was required to meet the threshold (see threshold section in “Methods”) depended on the body part and the threshold rule was applied after the image was adjusted for shadows and glares. A lower threshold was chosen for the head because the fraction of melanistic pattern area on the head was typically much higher (for example, see Figure S1, Panel A) so that the average pixel intensity was closer to the intensity of the melanistic spots. This lower threshold was effective for the head patterning, whereas it would not work effectively for the identifying the patterning of the other body parts, since the head patterning had a relatively high contrast between melanistic and non-melanistic regions.

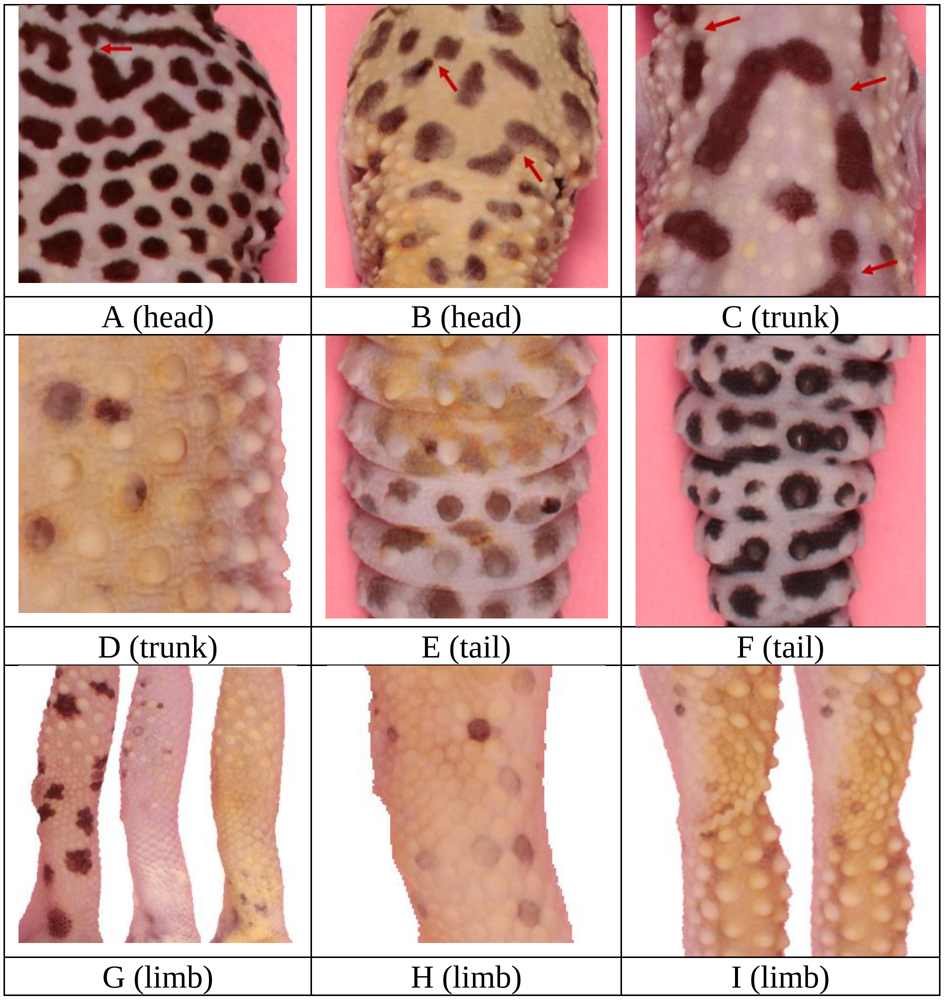

Figure S1: Representative images of patterns found on the four types of body parts; head (A,B) trunk (C, D), tail (E, F) and limb (G, H, I). Head and trunk patterns commonly had very high contrast between spotted and non-spotted pixels (A, C) though not always (B, D) and well-defined spots were often connected by a 'thread' with especially light pigmentation (red arrows). The head and trunk patterns often contained spots with a mixture of low and high eccentricity, with spots of both a small, rounded, compact type and a more elongated stripe-like type (A, C). While some patterns could be found with relatively even pigmentation throughout the spots, some patterns had spots with variable pigmentation within and/or between spots (B, D, E, H). Shadows and other types of light effects coincided with the pigmentation pattern due to the rounded contour of the leg (G) and the high protrusion of the tubercules on the trunk (D). Other pattern "defects" included occlusion from wrinkles of the skin that, for example, would appear on some of the replicates but not others (I)

Identification of interior and edge spots: Due to the fact that pictures were taken from above the geckos and due to the rounded shape of the gecko body, especially for the limbs, spots at the edges of the region were occasionally partially cut off from view. We call such spots whose boundary intersects with the edge of the body region "edge spots". Spots whose boundary lies

entirely within the region of the body in the image are called “interior spots” (see Figure A2 in the Appendix for an example). Some of our measures should be computed with the full contour of the spot, but are not affected if edge spots are removed, while other measures do not require the full contour of the spot, but are affected if edge spots are removed. For example, the fractional melanistic area of spots depends only on the total melanistic area, rather than the spot size and shapes. Other measures such as the average size and eccentricity of spots require the entire contour of a spot but do not depend on the total number of spots. For this reason, some measures were computed for the entire spot pattern, including both interior and edge spots, and some measures were computed only for the interior spots that were not cut off by the edge of the region (see Table 3).

Differences between “lemon frost” and "normal" morphs

Our data set contained 20 geckos of the "normal" and five of the “lemon frost” morphotypes. Although this sample size is relatively small for the “lemon frost” and as such, the analysis of this section should be regarded as preliminary, a clear qualitative difference in the patterns of “normal” and “lemon frost” morphotypes can clearly be detected by eye, so that it is maybe not so surprising that we still obtain several statistically significant differences even with these small samples. The difference between the “lemon frost” morph and the "normal" is illustrated in Figure 6 (right panel), where we show the first two principal components of the head, trunk and tail patterns of the two morphs. For both the head and the trunk patterns, but not the tail patterns, some clustering is observable with lemon frost geckos tending to have smaller values for both principal components. This corresponds to smaller spots arranged in patterns with a shorter wavelength. In fact, when we investigated the differences between the "normal" and the “lemon frost” morphs for the head and trunk patterns (TableS1) we found that these differences are statistically significant for the first two principal components of the head patterns and the second principal component for the trunk. For individual indices, the differences are statistically significant in a few cases, most notably those that are associated with the shape of the spots (EE and EL). In both cases, the “lemon frost” spots are closer to circular than the "normal" spots. In contrast to this, the differences in tail patterns were not statistically significant in any of the indices or principal components; the smallest p-value of all indices was 0.24. We also investigated whether one of the morphs showed more variation in traits than the other (Table S1). With some exceptions, the sample standard deviations of the “lemon frost” morph are smaller than those of the “normal” morph, meaning that the “lemon frost” morph tends to have smaller variability than the “normal” one. This is particularly true for the head and trunk patterns, but there is again relatively little difference between the variances of the tail. Most of these differences are not statistically significant due to the small sample size with only 5 “lemon frost” individuals, but for the trunk, both MD and PL are significantly smaller (along with 5 other indices or principal components), and for head, MD is significantly smaller as well (with 4 other variables also). Both MD and PL are measures of the characteristic wavelength of the pattern, i.e. the typical distance between spots. Thus our results indicate that the “lemon frost” morph has less variability in this measure than the “normal” one for the head and trunk.

Differences between sexes

Finally, we also investigated the differences between female and male patterning, restricting the analysis to the head patterns of individuals with “normal” morphs. We concentrated on the head patterns because these had the smallest measurement error. None of our indices showed any

significant differences (Table S2), consistent with the hypothesis that there is no significant difference between female and male melanistic patterns in captive bred individuals.

	FM	SS	SSD	EE	EED	PL	MD	MDD	SA	SAD	SI	SID	EL	ELD	PC1	PC2	PC3	PC4	PC5
**"Normal" Morph mean HD**	0.39	0.38	0.15	0.75	0.13	0.38	0.36	0.063	0.061	0.034	67	11	6	3.1	3.4	2.1	1.5	1.2	0.96
**"Lemon Frost" mean HD**	0.35	0.29*	0.13	0.69**	0.16**	0.46*	0.3*	0.055	0.038	0.023	69	12	5.3*	2.7	1.7*	0.66*	0.34*	0.55	0.42
**"Normal" Morph mean TR**	0.2	0.3	0.11	0.69	0.14	1.8	0.55	0.12	0.048	0.031	65	12	5.1	2.2	0.81	0.95	0.32	0.14	0.083
**"Lemon Frost" mean TR**	0.2	0.24	0.089	0.59**	0.15	1.2	0.32	0.058	0.035	0.022	62	11	4.1*	1.5	-0.23	-0.79*	-1.1*	-0.79	-0.61
**"Normal" Morph mean TA**	0.3	0.28	0.09	0.71	0.13	0.48	0.35	0.057	0.041	0.02	61	9.4	4.9	1.8	1.1	0.14	0.081	-0.013	-0.078
**"Lemon Frost" mean TA**	0.29	0.28	0.1	0.71	0.13	0.53	0.34	0.049	0.039	0.021	56	10	4.8	1.8	1.2	0.11	0.027	-0.052	-0.067
**std(LF HD) /std(Normal HD)**	0.54	0.44	0.62	1.5	1.9*	0.67	0.31*	0.42	0.24*	0.29*	0.36	1.7	0.7	0.96	0.48	0.68	0.67	0.71	0.69*
**std(LF TR) /std(Normal TR)**	0.55	0.79	0.82	0.93	1.1	0.18**	0.14**	0.24*	0.64	0.69	0.26*	0.34	0.69	0.52	0.68	0.58	0.55*	0.59*	0.61**
**std(LF TA) /std(Normal TA)**	0.52	0.58	1.4	1.2	1.4	1.3	0.42	0.19**	0.51	0.78	0.47	1	0.58	0.76	0.67	0.94	0.97	0.93	0.91

Table S1: First four rows: Mean values for all 14 indices and the first 5 principal components for the head, trunk and tail patterns of the "normal" and the “lemon frost” morphs. Stars in rows 2, 4 and 6 indicate the results of tests of significance of the hypothesis that the two mean are equal (two-sample t −test). Fifth and sixth row: Ratios of the sample standard deviations of the “lemon frost” morphs and the “normal” morphs. Stars indicate the results of tests of significance of the hypothesis that the two variances are equal (two-sample F-test for equal variances). One star (*) indicates p-values less than 0.05, ** p-values less than 0.01, *** p-values less than 0.001, **** p-values less than 0.00001.

	FM	SS	SSD	EE	EED	PL	MD	MDD	SA	SAD	SI	SID	EL	ELD	PC1	PC2	PC3	PC4	PC5
male ("normal" morph) HD	0.37	0.37	0.15	0.76	0.13	0.41	0.37	0.063	0.058	0.033	70	12	5.9	3.1	3.1	1.9	1.4	1.2	0.96
female ("normal" morph) HD	0.41	0.38	0.15	0.75	0.12	0.36	0.36	0.063	0.064	0.036	65	10	6.1	3.2	3.6	2.2	1.6	1.2	0.97
p-value(mean M= mean F)	0.1369	0.7936	0.8523	0.8096	0.2612	0.1895	0.6749	0.9822	0.6551	0.7155	0.4472	0.05769	0.562	0.755	0.5195	0.7144	0.7152	0.8488	0.9711

Table S2: Mean values for all 14 indices and the first 5 principal components for the head patterns of male and female "normal" morph geckos. The third row gives p-values for tests of significance of the hypothesis that the two mean are equal (two-sample t −test). Note that all p-values are well above 0.05, meaning that we cannot reject the hypothesis of equal means for any of the indices or principal components.

Supplementary Material: Data set of all body part images with binarizations and matlab code for image analysis and statistical analysis
