# Supplementary material for: Capturing and analyzing pattern diversity: an example using the melanistic spotted patterns of leopard geckos
Source: PeerJ. 2021 Sep 10;9:e11829. doi: 10.7717/peerj.11829 (PMC8436963; doi:10.7717/peerj.11829)
Supplement: Supplemental Information 3 — White: Mahalanobis distance. Gray: Developmental Noise distance. Distance squares are scaled so that the mean between-individual leg distance, i.e., the distance between two leg patterns of different individuals, is 1, see Fig. 7. Given are the ratios of these mean measurement errors to between-individual distances, or, for front legs and back legs, within-individual distances. One asterisk (*) indicates p-values less than 0.05, ** p-values less than 0.01, *** p-values less than 0.001, **** p-values less than 0.00001. [file peerj-09-11829-s003.docx]

**TABLE A1**
