# Supplementary material for: Capturing and analyzing pattern diversity: an example using the melanistic spotted patterns of leopard geckos
Source: PeerJ. 2021 Sep 10;9:e11829. doi: 10.7717/peerj.11829 (PMC8436963; doi:10.7717/peerj.11829)
Supplement: Supplemental Information 4 — Given are the F-values (mean square sums relative to the mean square sum of the error) of the factors “individuals”, “sides” and the interaction “sides <!–[if !msEquation]–> <!–[if !vml]–>x individuals”. Also given is the p-value for a t-test of the left versus right means. One asterisk (*) indicates p-values less than 0.05, ** p-values less than 0.01, *** p-values less than 0.001, **** p-values less than 0.00001. [file peerj-09-11829-s004.docx]

**TABLE A2**
