# Supplementary material for: Capturing and analyzing pattern diversity: an example using the melanistic spotted patterns of leopard geckos
Source: PeerJ. 2021 Sep 10;9:e11829. doi: 10.7717/peerj.11829 (PMC8436963; doi:10.7717/peerj.11829)
Supplement: Supplemental Information 5 — Weights of the first six principal components obtained on the 14 indices from all 132 data points corresponding to different geckos and body parts. Color intensity indicates the magnitude of the coefficients with positive values in red and negatives ones in blue. [file peerj-09-11829-s005.docx]

**TABLE A3**
