# Supplementary figures and images for: Capturing and analyzing pattern diversity: an example using the melanistic spotted patterns of leopard geckos
Source: PeerJ. 2021 Sep 10;9:e11829. doi: 10.7717/peerj.11829 (PMC8436963; doi:10.7717/peerj.11829)

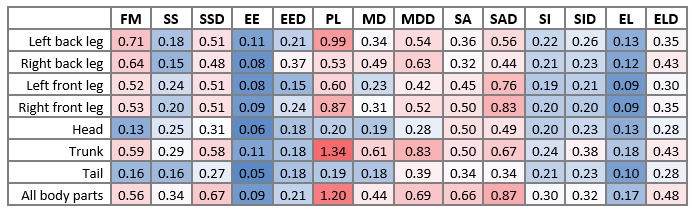


**TABLE A4**

Supplement: Supplemental Information 6 — Coefficients of variation (ratio of standard deviation and mean) for each of the 14 indices with values color coded from low (blue) to high (red). Rows: values for all body parts across all geckos; last row: values for all data points (geckos and body parts) combined. [file peerj-09-11829-s006.docx]

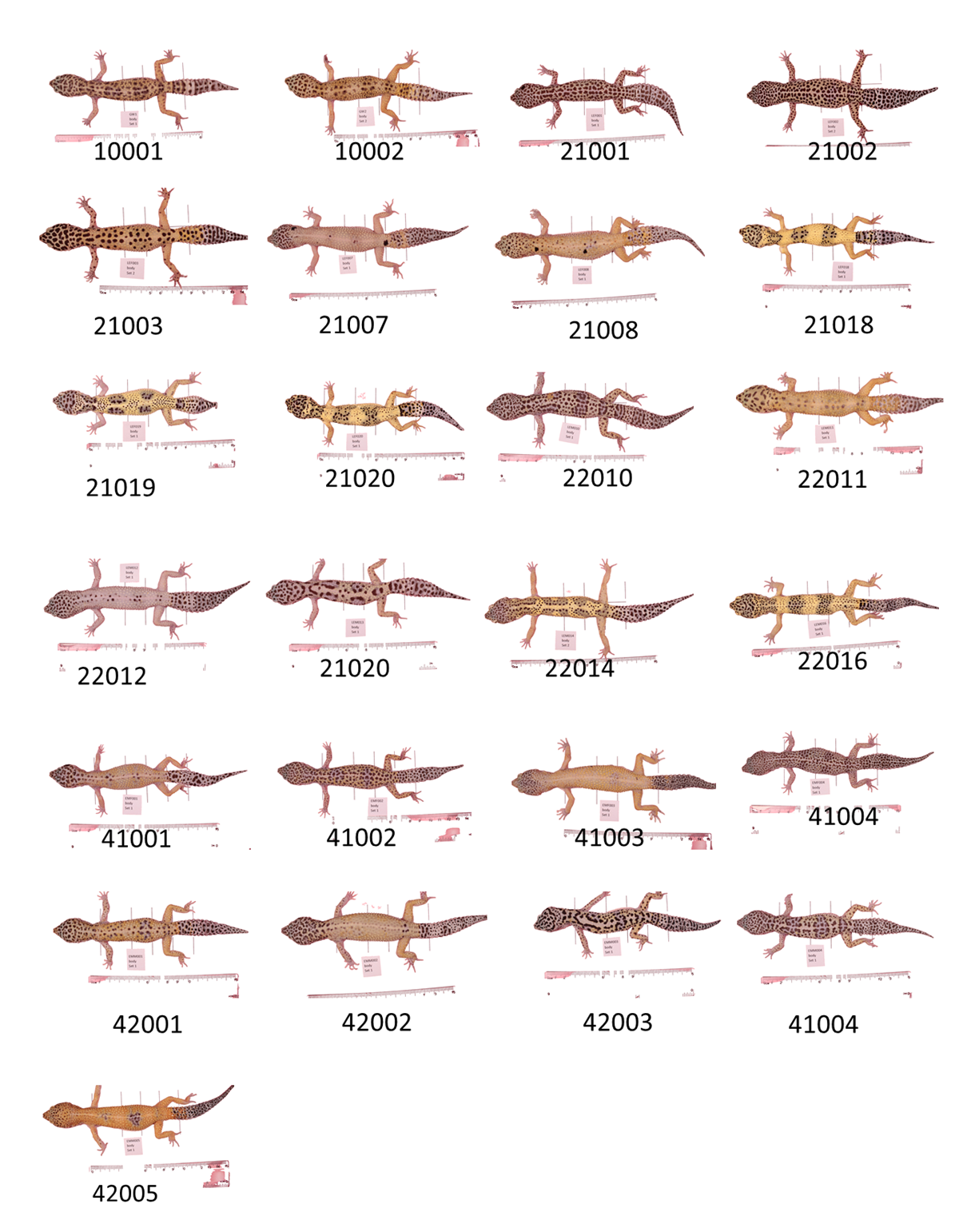

Supplement: Supplemental Information 7 [file peerj-09-11829-s007.png]

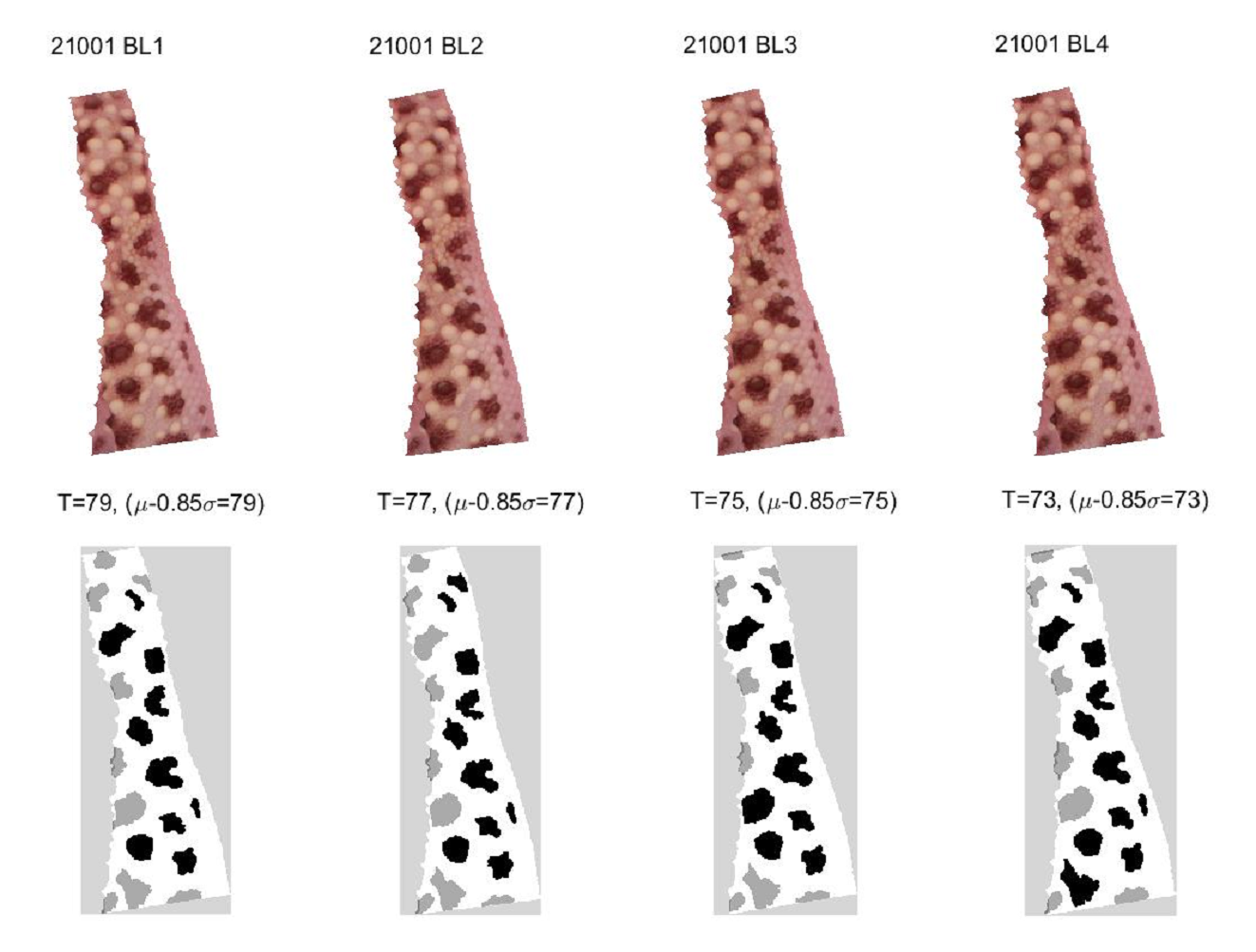

Supplement: Supplemental Information 8 — Example of the image binarization: Top: extracted photos of the left back leg of gecko # 21001 for four independent, repeated measurements. Bottom: Binarized image showing spots as detected by the spot detection algorithm for each repeated measurement. Black spots are interior spots, gray ones are boundary spots. The threshold for binarization T is determined by the average pixel value (intensity) (greek letter mu) and the standard deviation of pixel values (greek letter sigma) for the observed region of the limb (T = mu−0.85 sigma). [file peerj-09-11829-s008.png]

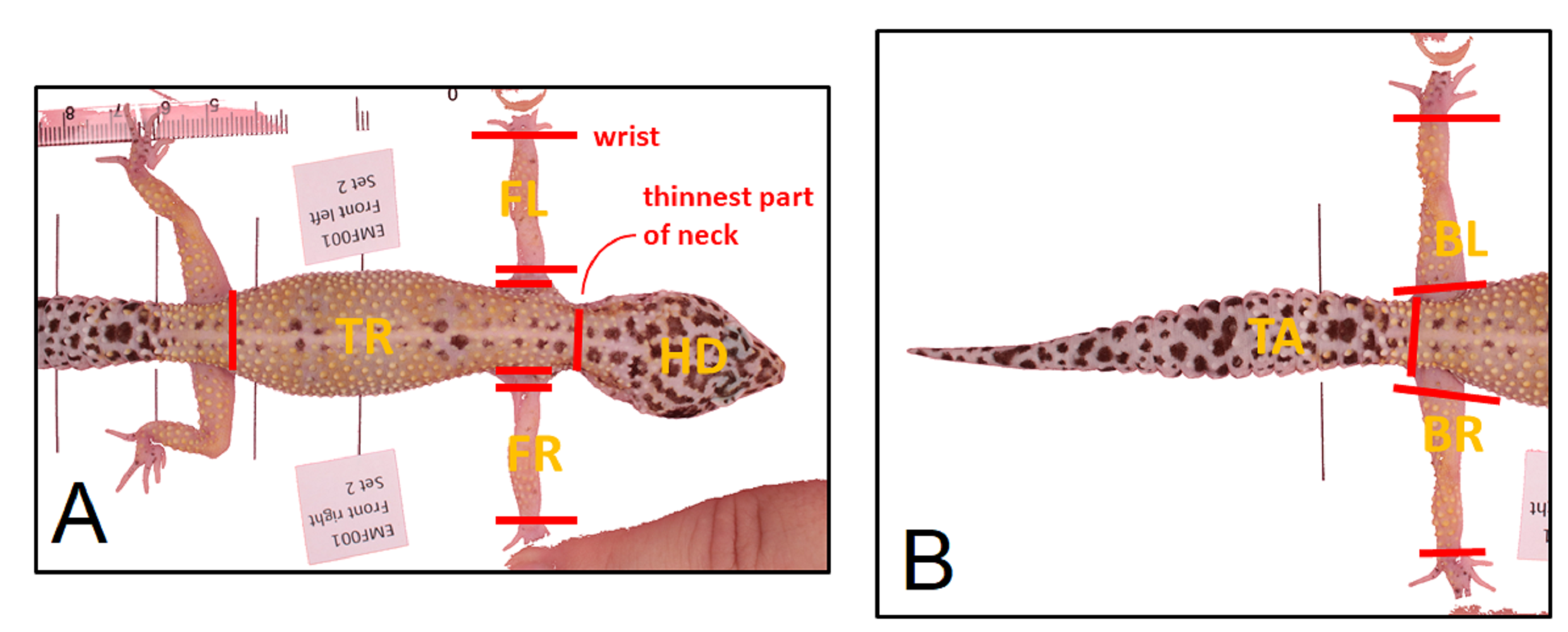

Supplement: Supplemental Information 9 — The white background was removed automatically with Matlab by removing pixels in a certain range of RGB values. The red cut were made manually for each image and indicates where boundaries of body parts are. [file peerj-09-11829-s009.png]
